# The Stability of Spiral-Grooved Air Journal Bearings in Ultrahigh Speeds

**DOI:** 10.3390/ma15051759

**Published:** 2022-02-25

**Authors:** Laiyun Song, Guoqin Yuan, Hongwen Zhang, Yalin Ding, Kai Cheng

**Affiliations:** Key Laboratory of Airborne Optical Imaging and Measurement, Changchun Institute of Optics, Fine Mechanics and Physics, Chinese Academy of Sciences, Changchun 130033, China; timsong1029@163.com (L.S.); yuanguoqin@139.com (G.Y.); dingyl_1964@126.com (Y.D.); kai.cheng@brunel.ac.uk (K.C.)

**Keywords:** spiral-grooved hybrid air journal bearings, stability of the air bearing, critical speed, high-speed condition, dynamic performance experiments

## Abstract

The spiral-grooved structure has been proposed for promoting the load capacity and stiffness of hybrid air journal bearings. In this paper, the dynamic characteristics of spiral-grooved hybrid bearings are first calculated. The stability criteria of the bearings are proposed and analyzed with different groove structure parameters using frequency domain analysis. It is found that the length of the spiral-groove has significant influence on the stability of the spindle system. Finally, the critical speed of the spiral-grooved hybrid bearing and rotor system is analyzed, and an experiment is carried out to validate the proposed model, finding that groove structure can promote the stability of the air bearing systems.

## 1. Introduction

In the field of optical lens processing, lens surface error is directly related to the spindle rotation error [1]. With the demand of high-speed and high-precision processing, the hybrid air bearings are widely applied in the high-speed and high precision spindles due to their inherent advantages: low friction, low vibration and low rotation error [2]. The hybrid air bearing is a special kind of air bearing in which the aerodynamic effect is equivalent with the aerostatic effect in high-speed conditions. However, the application of hybrid air bearings is limited due to instability, especially in high-speed conditions [3,4]. Thus, many researchers have studied the stability properties and proposed a solution to the instability problem in hybrid air journal bearings by optimizing the bearing parameters or designing a new structure [5,6,7].

There are two main ways to characterize the stability characteristics of the spindle: time domain analysis and frequency domain analysis. Fourka et al. [8] established a set of nonlinear theoretical models to analyze the stability of the aerostatic thrust bearings based on time domain analysis. Experiments were designed to compare and analyze the stability conditions of aerostatic thrust bearings, which proved the accuracy of the model. Miyatake et al. [9] analyzed the dynamic instability of a spindle system supported by a porous air-bearing radial bearing. A surface restriction technique was applied to improve the static stiffness of the air spindle and improve the dynamic stability of the spindle system at high speeds. Chen et al. [10] compared the stability of a spindle system with the orifices and inherences in the hybrid air bearing using the frequency domain analysis. The research showed that the stability of a spindle system with orifice is better than that with inherences at both high and low speeds. Maamari et al. [11] analyzed the dynamic performance and stability of a small-hole aerostatic bearing. Through a simulation analysis of fluid-solid coupling, a calculation model for comprehensive analysis of bearing stiffness and damping was established and verified by experiments. Zheng et al. [12] analyzed the air hammer instability problem of an aerostatic thrust bearing with pocket. The mathematical model established in the paper demonstrated that the main cause of air hammer is the delay effect of air flow.

To promote the load capacity and the stability of an air bearing-rotor system, many microstructures of air bearing were designed. To deal with the discontinuity of the grooves domain, researchers [13] applied the narrow groove theory (NGT) to obtain reasonable pressure distribution. Chen et al. [14] designed an X-shaped groove microstructure and studied the static performance and stability characteristics of the bearings using the resistance grid method (RNM). Studies have shown that this kind of X-shaped groove will enhance the bearing capacity of the bearing, but it will cause instability when the groove is too large or too deep. Belforte et al. [15] found that the existence of a pressure-equalizing groove enhances the bearing capacity and stiffness of the bearing, while the air consumption does not notably change. Yang et al. [16] further established a more accurate simulation model for aerostatic hemispherical bearings with spiral grooves, taking into account the movement of the spindle with 5 degrees of freedom. Stanev et al. [17] studied the influence of spiral grooves on high-speed aerostatic radial bearings and analyzed the influence of groove depth on dynamic and static performance and stability. However, the analysis on the effect of groove structure on the stability of the air bearings was limited and the influence of high speed was not mentioned in their study. Jia et al. [18] conducted a nonlinear dynamic analysis on a hemispherical aerostatic bearing with spiral groove using the Rouss–Horwitz stability criterion. The analysis results showed that the supply pressure, speed and eccentricity have notable influence on the dynamic performance of the bearing. Song et al. [19] numerically studied the dynamic characteristics of spiral-grooved hybrid air bearings and thoroughly analyzed the effect of groove structure parameters on the dynamic characteristics. The above study on a spiral-grooved hybrid air bearing focused mostly on the static and dynamic characteristics of air bearings, yet the study on the stability of spiral-grooved hybrid air journal bearings was limited, especially in high-speed conditions. Moreover, few studies examine the influence of groove structure parameters such as groove depth, groove length and groove angle on the stability of air bearings.

In this paper, the stability of spiral-grooved hybrid gas journal bearings in high- speed conditions is studied using frequency domain analysis. The stability characteristic is based on the dynamic stiffness and the damping coefficients, which is calculated by solving the unsteady Reynolds equation. The stability of a spiral-grooved hybrid air journal bearing is studied at different rotational speeds and eccentricity ratios. In addition, the influences of the groove parameters on the stability of the air bearings are discussed in detail. Finally, the critical speed is calculated under certain bearings and an experiment on the critical speed of the bearing-rotor system is conducted to verify the proposed numerical simulation.

## 2. Materials and Methods

### 2.1. Calculation of the Dynamic Characteristics of Spiral-Grooved Hybrid Air Journal Bearings

**Figure 1:**
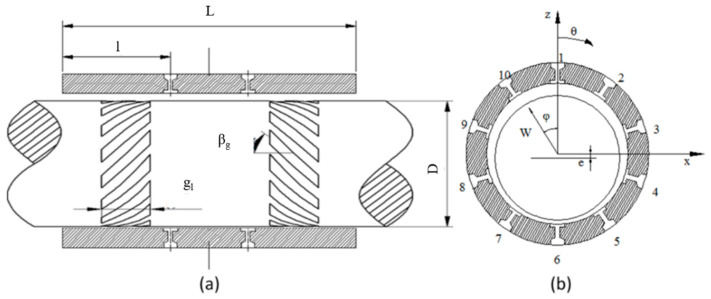
Schematic diagram of a spiral-grooved hybrid air bearing rotor system. (**a**) Circumferential section of the spiral grooved air spindle (β_g_ is the groove angle and g_l_ is the groove length) (**b**) Radial section of the spiral grooved air spindle.

**Figure 2:**
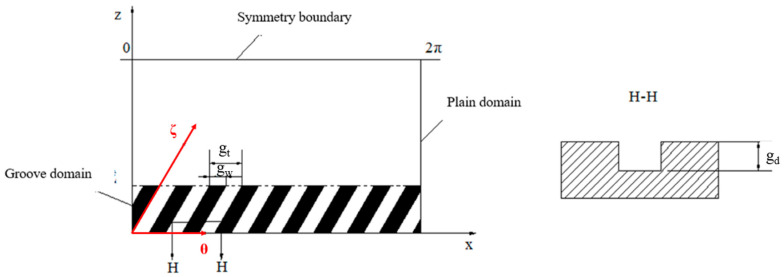
Calculation area of a grooved structure hybrid air bearing.

Figure 1 shows the structure of a spiral-grooved air bearing rotor system. Ten orifices in two rows are evenly distributed in the circumferential direction. The spiral grooves are also symmetrically distributed on the shaft. During high-speed rotation, these spiral grooves will pump air into the air film, thereby increasing the rigidity of the bearing. The calculation area is obtained by transforming the air film into a plane, as shown in Figure 2. The calculation domain is divided into a groove area and a plane area where the groove length is g_l_*,* the groove angle is β_g_*,* the groove depth is g_d_*,* the ratio of the groove width to the step width is g_t_/g_r_, and the number of grooves is g_n_.

In the plane area, the static pressure and the perturbation pressure are obtained by solving the unsteady Reynolds equation:

(1) $$\frac{\partial }{\partial X}\left(PH^{3}\frac{\partial P}{\partial X}\right)+\frac{\partial }{\partial Z}\left(PH^{3}\frac{\partial P}{\partial Z}\right)=\Lambda \frac{\partial (PH)}{\partial X}+2\Lambda \lambda \frac{\partial (PH)}{\partial \tau }$$

where Λ is the bearing number, *τ* is the dimensionless time where $\tau =tw_{s}$, *λ* is the perturbation ratio where $\lambda =w_{s}/w$, w_s_ is perturbation frequency of the spindle system.

To solve the unsteady Reynolds equation, the steady Reynolds equation and the perturbation equation should be solved together [19]. The static Reynolds equation can be denoted as:

(2) $$\frac{\partial }{\partial X}\left(P_{0}H_{0}{}^{3}\frac{\partial P_{0}}{\partial X}\right)+\frac{\partial }{\partial Z}\left(P_{0}H_{0}{}^{3}\frac{\partial P_{0}}{\partial Z}\right)=\Lambda \frac{\partial (P_{0}H_{0})}{\partial X}$$

The perturbation equation can be denoted as (Δ*x* for example):

(3) $$\begin{array}{l} \frac{\partial }{\partial X}\left(P_{0}H_{0}{}^{3}\frac{\partial P_{X}}{\partial X}+H_{0}{}^{3}\frac{\partial P_{0}}{\partial X}P_{X}+3P_{0}H_{0}{}^{2}\frac{\partial P_{0}}{\partial X}H_{X}\right)\\ +(\frac{R^{2}}{L^{2}})\frac{\partial }{\partial Z}\left(P_{0}H_{0}{}^{3}\frac{\partial P_{X}}{\partial Z}+H_{0}{}^{3}\frac{\partial P_{0}}{\partial Z}P_{X}+3P_{0}H_{0}{}^{2}\frac{\partial P_{0}}{\partial Z}H_{X}\right)\\ =\Lambda \frac{\partial (P_{0}H_{X}+H_{0}P_{X})}{\partial X}-2\Lambda \lambda (H_{0}P_{XX}+P_{0}H_{XX}) \end{array}$$

where P_0_ and H_0_ are the static dimensionless pressure and the static dimensionless film thickness, P_x_ and H_x_ are the first-order perturbation pressure and the first-order perturbation film thickness, P_xx_ and H_xx_ are the second-order perturbation pressure and the second-order perturbation film thickness, respectively.

In the groove area, the coordinate system needs to be changed for the groove structure. As shown in Figure 2, the original x-z plane is transformed into the *θ*-*ζ* plane:

(4) $$\begin{array}{l} X=\frac{Z}{\tan\beta_{g}}+\theta \\ Z=\zeta \sin\beta_{g} \end{array}$$

Thus, the steady Reynolds equation in the grooved domain can be transformed into:

(5) $$\begin{array}{l} (1+\frac{R^{2}}{L^{2}}\frac{1}{\tan^{2}\beta_{g}})\frac{\partial }{\partial \theta }\left(P_{0}H_{0}{}^{3}\frac{\partial P_{0}}{\partial \theta }\right)+\frac{1}{\sin^{2}\beta_{g}}\frac{R^{2}}{L^{2}}\frac{\partial }{\partial \xi }\left(P_{0}H_{0}{}^{3}\frac{\partial P_{0}}{\partial \xi }\right)\\ =\Lambda \frac{\partial (P_{0}H_{0})}{\partial \theta }+\frac{1}{\tan\beta_{g}\sin\beta_{g}}\frac{R^{2}}{L^{2}}(\frac{\partial }{\partial \theta }\left(P_{0}H_{0}{}^{3}\frac{\partial P_{0}}{\partial \xi }\right)+\frac{\partial }{\partial \xi }\left(P_{0}H_{0}{}^{3}\frac{\partial P_{0}}{\partial \theta }\right)) \end{array}$$

The perturbation equations can be transformed into (Δ*x* for example):

(6) $$\begin{array}{l} (1+\frac{R^{2}}{L^{2}}\frac{1}{\tan^{2}\beta_{g}})\frac{\partial }{\partial \theta }\left(P_{0}H_{0}{}^{3}\frac{\partial P_{X}}{\partial \theta }+H_{0}{}^{3}\frac{\partial P_{0}}{\partial \theta }P_{X}+3P_{0}H_{0}{}^{2}\frac{\partial P_{0}}{\partial \theta }H_{X}\right)\\ +\frac{1}{\sin^{2}\beta_{g}}\frac{R^{2}}{L^{2}}\frac{\partial }{\partial \xi }\left(P_{0}H_{0}{}^{3}\frac{\partial P_{X}}{\partial \xi }+H_{0}{}^{3}\frac{\partial P_{0}}{\partial \xi }P_{X}+3P_{0}H_{0}{}^{2}\frac{\partial P_{0}}{\partial \xi }H_{X}\right)=\Lambda \frac{\partial (P_{0}H_{0}+H_{0}P_{X})}{\partial \theta }\\ +\frac{1}{\tan\beta_{g}\sin\beta_{g}}\frac{R^{2}}{L^{2}}(\frac{\partial }{\partial \theta }\left(P_{0}H_{0}{}^{3}\frac{\partial P_{X}}{\partial \xi }+H_{0}{}^{3}\frac{\partial P_{0}}{\partial \xi }P_{X}+3P_{0}H_{0}{}^{2}\frac{\partial P_{0}}{\partial \xi }H_{X}\right)\\ +\frac{\partial }{\partial \xi }\left(P_{0}H_{0}{}^{3}\frac{\partial P_{X}}{\partial \theta }+H_{0}{}^{3}\frac{\partial P_{0}}{\partial \theta }P_{X}+3P_{0}H_{0}{}^{2}\frac{\partial P_{0}}{\partial \theta }H_{X}\right))-2\Lambda \lambda (H_{0}P_{\mathrm{xx}}+P_{0}H_{\mathrm{xx}}) \end{array}$$

The above equations are discretized using the finite difference method(FDM). Thus, the static pressure in the grooved area can be depicted as:

(7) $$P_{0i,j}=\sqrt{\frac{\begin{array}{l} A_{i,j}^{t}P_{0i,j+1}^{2}+B_{i,j}^{t}P_{0i,j-1}^{2}+C_{i,j}^{t}P_{0i+1,j}^{2}+D_{i,j}^{t}P_{0i-1,j}^{2}-\Lambda \Delta \theta (P_{0i,j+1}H_{0i,j+1}-P_{0i,j-1}H_{0i,j-1})\\ -(F_{i,j}^{t}P_{0i+1,j+1}^{2}+G_{i,j}^{t}P_{0i-1,j+1}^{2}+I_{i,j}^{t}P_{0i+1,j-1}^{2}+J_{i,j}^{t}P_{0i-1,j-1}^{2}) \end{array}}{E_{i,j}^{t}}}$$

The perturbation pressure (Δ*x* for example) in the grooved area can be depicted as:

(8) $$\begin{array}{l} P_{Xi,j}=(A^{{}^{\prime }t}{}_{i,j}P_{Xi,j+1}+B^{{}^{\prime }t}{}_{i,j}P_{Xi,j+1}+C^{{}^{\prime }t}{}_{i,j}P_{Xi,j+1}+D^{{}^{\prime }t}{}_{i,j}P_{Xi,j+1}-F^{{}^{\prime }t}{}_{i,j}-Q_{i,j}^{{}^{\prime }t}P_{XXi,j}\\ +G^{{}^{\prime }t}{}_{i,j}P_{Xi+1,j+1}+I^{{}^{\prime }t}{}_{i,j}P_{Xi-1,j+1}+J^{{}^{\prime }t}{}_{i,j}P_{Xi+1,j1}+K^{{}^{\prime }t}{}_{i,j}P_{Xi-1,j-1})/E^{{}^{\prime }t}{}_{i,j} \end{array}$$

The static pressure and the perturbation pressure in the plain area and the coefficients A ^t^_i,j_~J^t^_ij_ and A ^t^′_i,j_~K ^t^′_ij_ can be obtained in the previous reference [19]. It is noted that the equation of perturbation pressure P_x_ and P_xx_ is coupled, which should be solved together. The convergence criterion in the present study is:

(9) $$\sum_{j=1}^{nx+1}\sum_{i=1}^{nz+1}\left|P_{Xi,j}^{k}-P_{Xi,j}^{k-1}\right|<10^{-6},\sum_{j=1}^{nx+1}\sum_{i=1}^{nz+1}\left|P_{XXi,j}^{k}-P_{XXi,j}^{k-1}\right|<10^{-6}$$

Finally, the dynamic performance of the bearing is calculated through P_x_, P_xx_, P_y_ and P_yy_. The dynamic stiffness and damping coefficients are:

(10) $$\left(\begin{array}{l} kxx\\ kyx \end{array}\right)=\frac{P_{a}RL}{h_{m}}\int_{0}^{1}\int_{0}^{2\pi }P_{X}\left(\begin{array}{l} \sin\varphi \\ -\cos\varphi \end{array}\right)d\varphi dZ$$

(11) $$\left(\begin{array}{l} kxy\\ kyy \end{array}\right)=\frac{P_{a}RL}{h_{m}}\int_{0}^{1}\int_{0}^{2\pi }P_{Y}\left(\begin{array}{l} \sin\varphi \\ -\cos\varphi \end{array}\right)d\varphi dZ$$

(12) $$\left(\begin{array}{l} dxx\\ dyx \end{array}\right)=\frac{P_{a}RL}{wh_{m}}\int_{0}^{1}\int_{0}^{2\pi }P_{XX}\left(\begin{array}{l} \sin\varphi \\ -\cos\varphi \end{array}\right)d\varphi dZ$$

(13) $$\left(\begin{array}{l} dxy\\ dyy \end{array}\right)=\frac{P_{a}RL}{wh_{m}}\int_{0}^{1}\int_{0}^{2\pi }P_{YY}\left(\begin{array}{l} \sin\varphi \\ -\cos\varphi \end{array}\right)d\varphi dZ$$

### 2.2. Stability Characteristics of Spiral-Grooved Hybrid Air Journal Bearings

Based on the calculated dynamic characteristics of spiral-grooved hybrid air journal bearings, the stability characteristics can be depicted by frequency domain analysis as follows:

First, it can be assumed that the shaft rotates round the equilibrium position with the frequency of w_s_. The dimensionless motion control equation under the x-y coordinates is:

(14) $$[\begin{array}{l} \bar{M}\lambda^{2}\\ 0 \end{array}\begin{array}{l} 0\\ \bar{M}\lambda^{2} \end{array}]\left[\begin{array}{l} \overset{\cdot \cdot }{\bar{x}}\\ \overset{\cdot \cdot }{\bar{y}} \end{array}\right]+[\begin{array}{l} \lambda \bar{B_{xx}}\\ \lambda \bar{B_{yx}} \end{array}\begin{array}{l} \lambda \bar{B_{xy}}\\ \lambda \bar{B_{yy}} \end{array}]\left[\begin{array}{l} \overset{\cdot }{\bar{x}}\\ \overset{\cdot }{\bar{y}} \end{array}\right]+[\begin{array}{l} \bar{K_{xx}}\\ \bar{K_{yx}} \end{array}\begin{array}{l} \bar{K_{xy}}\\ \bar{K_{yy}} \end{array}]\left[\begin{array}{l} \overset{}{\bar{x}}\\ \overset{}{\bar{y}} \end{array}\right]=\left[\begin{array}{l} 0\\ 0 \end{array}\right]$$

where $\bar{M}$ is the dimensionless mass where $\bar{M}=\frac{mcw^{2}}{2P_{a}LD}$.

The perturbation motion of the shaft can be denoted as:

(15) $$\begin{array}{l} \bar{x}=\bar{X}e^{st}\\ \bar{y}=\bar{Y}e^{st} \end{array}$$

where $\bar{X}$,$\bar{Y}$ is the dimensionless amplitude, s is the oscillation coefficient, which is in plural form, $s=(c_{\beta }/\Omega )\pm i$, c*_β_* is the damping variable and Ω is perturbation frequency.

In the critical state, we have:

(16) $$\begin{array}{l} c_{\beta }=0\\ \lambda =\lambda_{c}=\Omega /w_{c} \end{array}$$

By substituting Equations (15) and (16) in Equation (14):

(17) $$[\begin{array}{l} \bar{K_{xx}}+i\lambda_{c}\bar{B_{xx}}-\bar{M}\lambda_{c}{}^{2}\\ \bar{K_{yx}}+i\lambda_{c}\bar{B_{yx}} \end{array}\begin{array}{l} \bar{K_{xy}}+i\lambda_{c}\bar{B_{xy}}\\ \bar{K_{xx}}+i\lambda_{c}\bar{B_{xx}}-\bar{M}\lambda_{c}{}^{2} \end{array}]\left[\begin{array}{l} \bar{X}\\ \bar{Y} \end{array}\right]=\left[\begin{array}{l} 0\\ 0 \end{array}\right]$$

There is a nonzero solution of Equation (17). It can be obtained:

(18) $$\begin{array}{l} I_{c}=\frac{\bar{K_{xx}}\bar{B_{x}}+\bar{K_{yy}}\bar{B_{xx}}-\bar{K_{xy}}\bar{B_{yx}}-\bar{K_{yx}}\bar{B_{xy}}}{\bar{B_{xx}}+\bar{B_{yy}}}\\ \lambda_{c}{}^{2}=\frac{(I_{c}-\bar{K_{xx}})(I_{c}-\bar{K_{yy}})-\bar{K_{xy}}\bar{K_{yx}}}{\bar{B_{xx}}\bar{B_{yy}}-\bar{B_{xy}}\bar{B_{yx}}} \end{array}$$

where I_c_ is the critical mass and *λ*_c_ is the critical perturbation ratio. It is noted that the dynamic characteristics of the air bearing system is the function of the perturbation ratio *λ*. As a result, the critical perturbation ratio should be solved iteratively.

**Figure 3:**
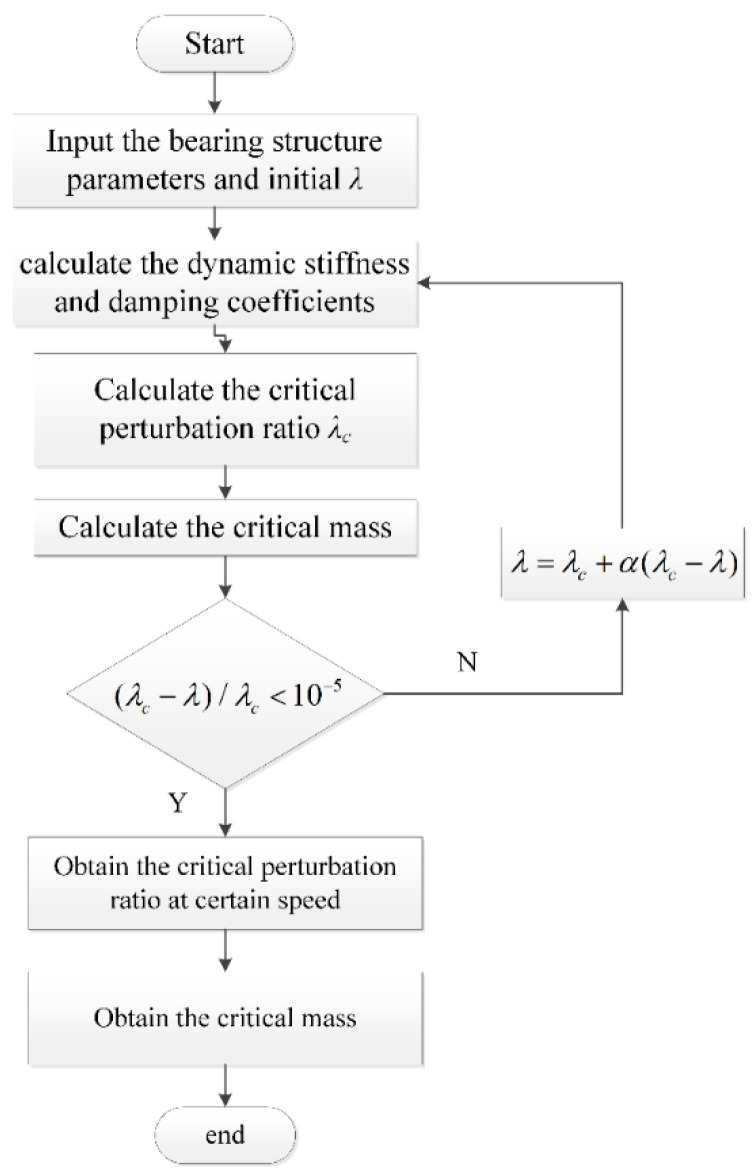
The process of obtaining the static and dynamic performances of gas journal bearings.

In this study, to obtain the stability characteristics of the grooved hybrid air journal bearing rotor system, the unsteady Reynolds equations with initial perturbation ratios are first solved to calculate the dynamic stiffness and damping coefficients. Then, the critical perturbation ratio is calculated iteratively until the critical perturbation ratio equals the previous perturbation ratio at the certain speed. The flowchart of numerical calculation is shown in Figure 3. The structure parameters of the grooved hybrid air journal bearing are listed in Table 1.

## 3. Results

**Figure 4:**
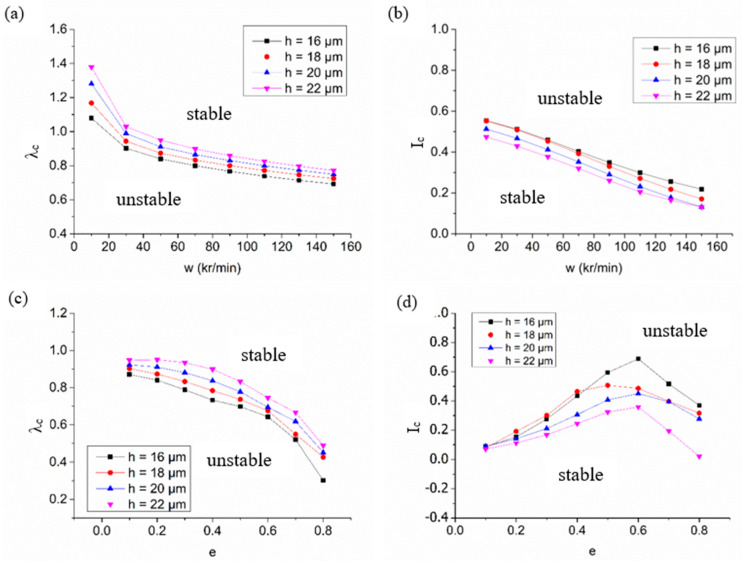
The stability characteristics of hybrid air bearings with variations of working conditions with different film thicknesses: (**a**) variation of critical perturbation ratio with speed; (**b**) variation of critical mass with speed; (**c**) variation of critical perturbation ratio with eccentricity ratio; (**d**) variation of critical mass with eccentricity ratio.

Figure 4 shows the stability performance of a spiral-grooved hybrid air bearing system with different air film thicknesses in variation of the rotation speed and the eccentricity ratio. In general, a system with larger critical mass and smaller critical perturbation ratio gives larger stability margin. Thus, the domain above the critical mass and regions below the critical perturbation frequency are unstable. As Figure 4a depicts, the perturbation frequency ratio decreases with increment of rotation speed, which means the spiral-grooved hybrid air journal bearings obtain better stability in high-speed conditions. It can also be seen that a smaller gas film thickness can obtain a smaller perturbation ratio in the full-speed range, which can increase the stability of the system because a small air film thickness will result in the increment dynamic stiffness and dynamic damping of the system. For the air bearing system with high eccentricity, this phenomenon is more obvious, as shown in Figure 4c. A small air film thickness will decrease the critical perturbation ratio of the system, thereby improving the stability of the rotor system. In terms of critical mass, the critical mass also declines with the growing rotation speed, as shown in Figure 4b. As Figure 4d shows, the critical mass grows first and decreases with the increment of the eccentricity ratio. As a result, the mass design should be careful to reduce the shaft mass in high-speed conditions.

**Figure 5:**
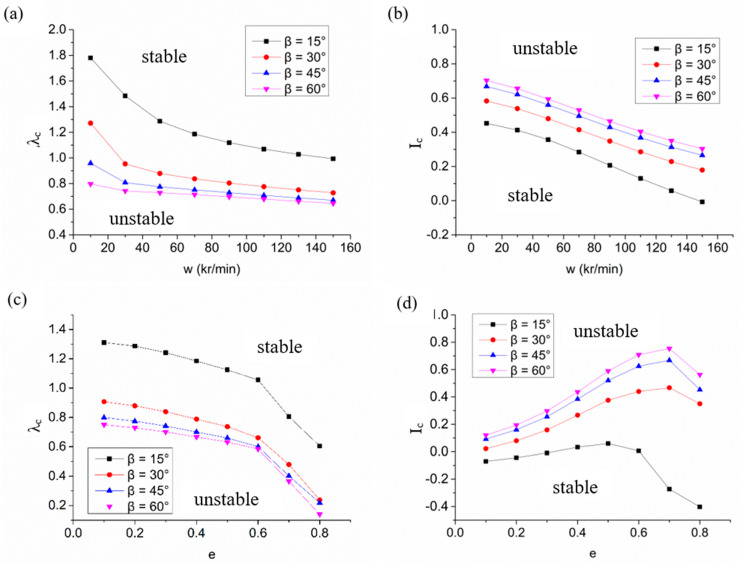
The stability characteristics of hybrid air bearings with variations of working conditions with different groove angles: (**a**) variation of critical perturbation ratio with speed; (**b**) variation of critical mass with speed; (**c**) variation of critical perturbation ratio with eccentricity ratio; (**d**) variation of critical mass with eccentricity ratio.

Figure 5 illustrates the effect on the stability of a spiral-grooved hybrid air bearing-spindle system when the groove angle is 15°, 30°, 45° and 60°. When eccentricity is 0.2 under the full-speed range, the air bearing system with the larger groove angle obtains a smaller critical perturbation ratio and greater critical mass, which leads to better system stability. However, when the groove angle increases from 45° to 60°, the enhanced effect in stability is limited, as shown in Figure 5a,b. Similarly, under different eccentricities, the stability of the groove helix angle between 45° and 60° is better than that of the small groove angle because the increase of the groove angle has no effect on the dynamic damping of the hybrid air bearing, but the dynamic stiffness of the bearing is improved, thereby improving the stability of the spindle.

**Figure 6:**
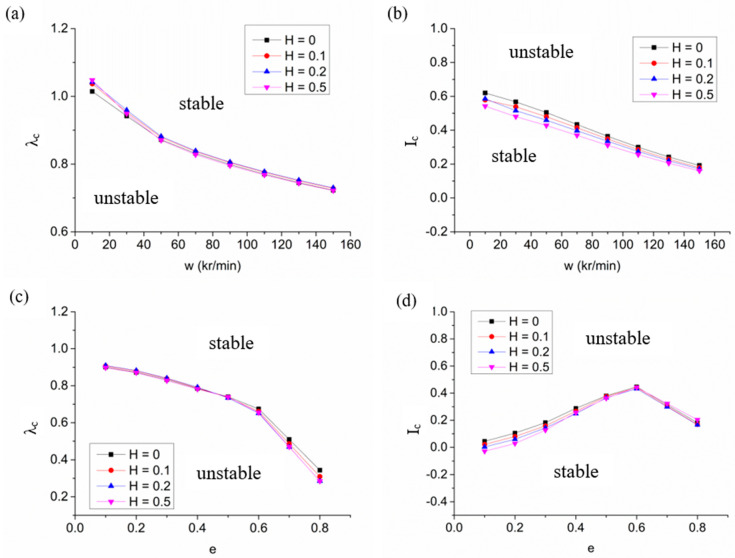
The stability characteristics of hybrid air bearings with variations of working conditions with different groove depths: (**a**) variation of critical perturbation ratio with speed; (**b**) variation of critical mass with speed; (**c**) variation of critical perturbation ratio with eccentricity ratio; (**d**) variation of critical mass with eccentricity ratio.

According to the reference [19], it can be verified that too large a groove depth will cause the damping of the bearing to drop sharply. Therefore, the groove depth ratio studied in this section is limited to 0, 0.1, 0.2 and 0.5. As shown in Figure 6, the influence of groove depth on the stability of hybrid air bearings is very limited. When the eccentricity is 0.2, at full speed, increasing the groove depth of the microstructure groove has almost no effect on the critical perturbation ratio of the system, but will slightly reduce the critical mass of the rotor. Under high eccentricity, the critical perturbation ratio decreases with the growth of the groove depth because the groove depth mainly affects the cross dynamic stiffness and cross dynamic damping of the air bearing. Although the groove depth increases the damping coefficient of the bearing system, it also improves the cross stiffness of the system. Comprehensively, the increase in groove depth will only slightly improve the stability of the spindle system.

**Figure 7:**
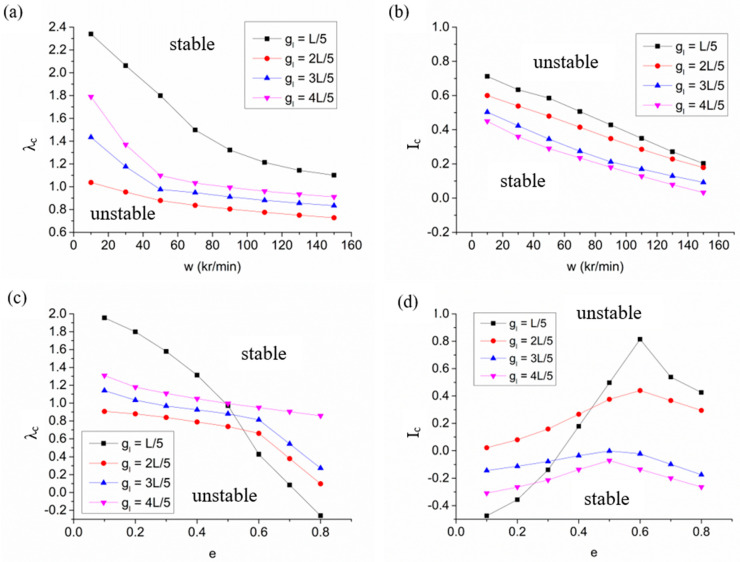
The stability characteristics of hybrid air bearing with variations in working conditions with different groove length: (**a**) variation of critical perturbation ratio with speed; (**b**) variation of critical mass with speed; (**c**) variation of critical perturbation ratio with eccentricity ratio; (**d**) variation of critical mass with eccentricity ratio.

Figure 7 demonstrates the law of influence on the stability of a hybrid air bearing-spindle system when the groove length is L/5, 2L/5, 3L/5 and 4L/5. As Figure 7a,b depict, with the continuous increase in speed, the stability of the air spindle system is sensitive to the influence of groove length. Although a shorter groove length can improve the critical mass of the rotor, the critical perturbation ratio of the system increases, and the system is prone to instability. A longer groove length will not only reduce the critical mass, but also increase the critical perturbation ratio of the spindle, thus greatly reducing the stability of the system. Therefore, choosing a moderate slot length (2/5L in this study) can improve the critical mass of the rotor and obtain better bearing system stability. In addition, in the full eccentricity range, the appropriate groove length will maintain the critical perturbation ratio at a very low level, and the spindle stability is high. Too short Groove length that is too short easily causes instability under low eccentricity, as shown in Figure 7c,d.

**Figure 8:**
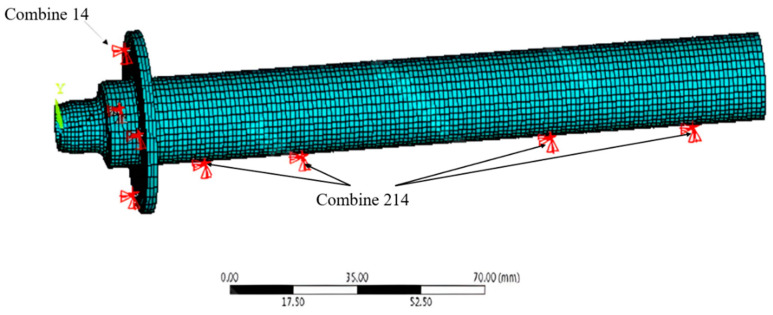
Equivalent unit of film rigidity of hybrid air bearing.

The finite element model of the hybrid air spindle system is shown in Figure 8. The shaft is meshed by a three-dimensional 20-node Solid186 element, and the axial bearing is substituted by linear Combine14, which are evenly distributed in the axial direction. Considering the dynamic stiffness and damping coefficients of the radial bearing with two degrees of freedom, the radial bearing is substituted by Combine214 for equivalency. The bearing position is at the double-row orifices of a spiral-grooved hybrid air bearing.

The natural frequencies of a spiral-grooved hybrid air spindle at different speeds are shown in Table 2. The first-order natural frequency of the spindle is 0 Hz, and the vibration mode is the rotation of the spindle with unrestricted degrees of freedom. As the shaft is slender type, the second- to fourth-order vibration modes are yaw around the Y axis, the front end of the shaft around the X axis and the rear end of the shaft around the X axis, respectively. The corresponding natural frequencies are 617.67 Hz, 1697.6 Hz, and 2148.8 Hz, respectively. The above three natural frequencies are the main vibration sources of the spiral-grooved hybrid air spindle. The fifth-order natural frequency is 3480.8 Hz, which corresponds to axial reciprocating motion. The sixth- and seventh-order frequencies correspond to the bending vibration mode of the shaft, and the eighth-order frequency corresponds to the torsional vibration mode of the shaft. The fifth to eighth natural frequencies are above 4000 Hz, which far exceed the movement frequency of the spindle system. In addition, the table shows that as the speed increases, the natural frequency of the second order remains unchanged, and the natural frequencies of the third order to the fifth order increase. This is because the yaw around the Y-axis corresponding to the second order is mainly determined by the axial stiffness, while the yaw around the X-axis at the front and rear ends of the shaft is mainly determined by the radial stiffness.

**Figure 9:**
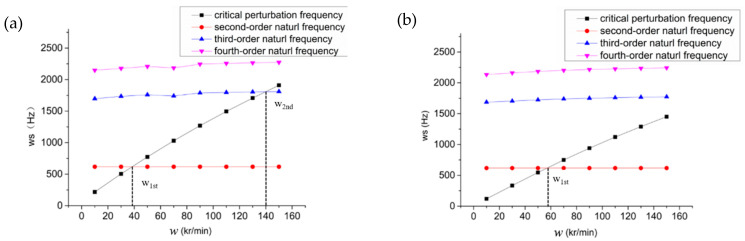
Critical speed of hybrid air bearing-rotor system. w_s_: critical perturbation frequency; w_1st_: first critical rotational speed of the aerostatic spindle system; w_2nd_: first critical rotational speed of the aerostatic spindle system; (**a**) without spiral grooves; (**b**) with spiral grooves.

Finally, the critical speed of the hybrid air spindle is calculated and analyzed both with and without spiral grooves, as shown in Figure 9. For the hybrid air bearing without grooves, the first-order critical speed is the speed when the critical perturbation frequency is equal to the second-order free frequency of the system, about 40,000 r/min. The second-order critical speed is the speed when the system’s critical perturbation frequency is equal to the second-order free frequency, about 140,000 r/min. For the hybrid air bearings with grooves, the first-order critical speed is about 58,000 r/min. The maximum design speed of the system is 150,000 r/min. In this speed range, the system frequency is less than the second-order natural frequency, so the second-order critical speed cannot be reached.

Table 3 shows the dynamic stiffness, damping coefficients, critical perturbation frequency, critical mass and critical velocity of the hybrid air bearing with spiral groove microstructure under different eccentricities (loads). The table shows that compared with the ordinary air bearing rotor system, the spiral groove microstructure improves the dynamic stiffness and damping coefficients of the hybrid air bearing and reduces the critical perturbation frequency of the system, thereby increasing the first-order critical speed.

## 4. Experiments

Based on the theoretical study of dynamic stability in Section 3, a dynamic vibration test was carried out for the developed high-speed hybrid air spindle both with and without spiral grooves. For the hybrid air spindle working at a certain rotational speed, the system energy increased, and the vibration was aggravated if the rotational speed was near the critical speed of the system. Therefore, the stability of the hybrid air bearing rotor system at each speed could be qualitatively analyzed by measuring the vibration of the spindle system.

**Figure 10:**
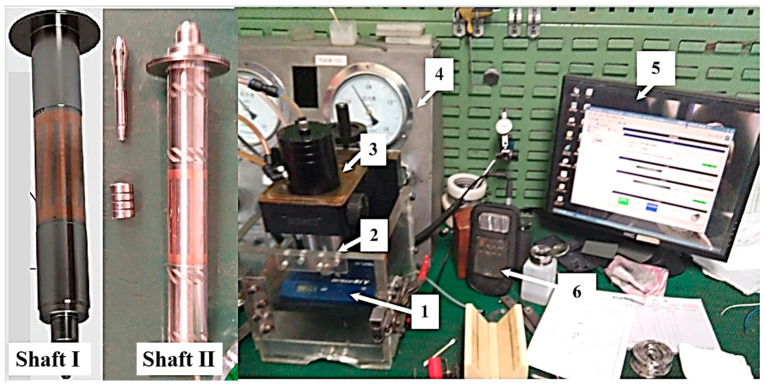
High-speed hybrid air spindle vibration experiment device: (1) spindle runout tester; (2) hybrid air spindle; (3) test bench; (4) air filter; (5) rotor speed control software; (6) spindle vibration measuring instrument.

The measured shafts were ordinary shaft I and shaft II with spiral grooves, as shown in Figure 10. The rotation speed of the spindle was controlled by software, and the vibration of the spindle was tested by a measuring instrument, which was a dual-channel vibration accelerometer measuring instrument, KMbalance Ⅱ. The measurement sensitivity was 8.09 mV/g. The rotational speed was measured by the speed sensor at the end of the rotor.

**Figure 11:**
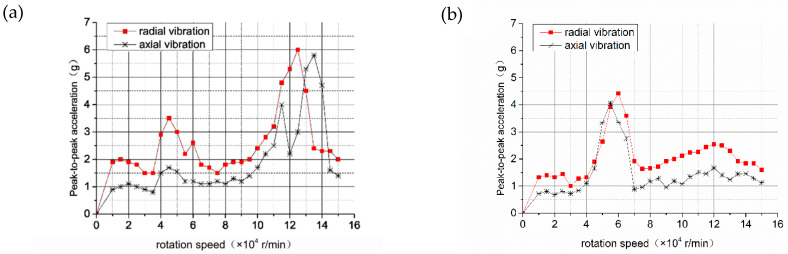
Axial and radial acceleration peak-to-peak changes of shaft I and shaft II with speed: (**a**) vibration peak-to-peak value of shaft Ⅰ; (**b**) vibration peak-to-peak value of shaft Ⅱ.

The experiment measured the peak-to-peak acceleration of the radial and axial vibration of shaft I and shaft II at different speeds, as shown in Figure 11. It is known that the rotational speed when the peak value of the vibration occurs is the critical speed of the aerostatic spindle system. Figure 11 shows the radial vibration peaks of shaft I appeared at speeds 42,500 r/min and 13,7000 r/min. The peak value of axial vibration was 143,000 r/min. Table 4 illustrates the difference between the numerical model and the experiment result of the critical speed for both shaft I and shaft II. The table verifies the numerical model and the experiment results are consistent, validating the numerical model with the critical speed analysis of the high-speed hybrid air bearing rotor system, with less than a 10% error.

Comparing Figure 11a,b, it can be seen that the spiral groove microstructure optimizes dynamic stiffness and dynamic damping of the hybrid air bearing, which reduces the overall vibration of the system in the full speed range, and the vibration peak appears at 54,200 r/min, which is consistent with the critical speed calculated in Section 3. Therefore, the vibration experiment of the high-speed hybrid air spindle proves the validity of the calculation model on the stability and critical speed of the hybrid air bearing-rotor system.

By comparing the high-speed hybrid air spindle developed in the literature [19], this study reduced the average peak-to-peak vibration value from 4.5 g to 4.1 g by optimizing the groove parameters of the bearing, and the vibration value decreased by 8.9%.

## 5. Conclusions

This paper has established the stability model of a spiral-grooved hybrid air bearing rotor system in high-speed conditions based on the dynamic performance results of hybrid air bearings. The stability criterion of a spiral-grooved hybrid air spindle system was proposed, and the critical perturbation ratio and the critical mass of the spindle system were analyzed in terms of the groove structure parameters. Furthermore, the critical speed was predicted under certain groove parameters by a FDM model of the rotor-bearing systems. Finally, the predicted critical speed was verified by a designed experiment that measured the vibration of the hybrid air spindle both with and without spiral grooves. The following conclusions can be drawn:

1. With increased rotation speed, the critical perturbation ratio increases, while the critical mass decreases. It can be concluded that the stability margin is enhanced by increasing rotation speed.
2. The stability margin of a spiral-grooved hybrid air spindle can be promoted by enlarging the groove angle. However, when the groove angle is larger than 45°, the enhancement is not obvious. The influence of groove depth on stability characteristics is limited because groove depth mainly affected the cross-coupled stiffness. The hybrid air spindle with spiral grooves that are too large or too short will obtain a lower critical perturbation ratio, which leads to a decline in stability.
3. The second- to fourth-order natural frequencies of a hybrid air spindle are the main causes of spindle vibration, which are responsive to yaw around the *Y* axis, to the front end of the shaft around the *X* axis and to the rear end of the shaft around the *X* axis, respectively. The critical speed of a hybrid air spindle with spiral grooves is larger than that of a spindle without spiral grooves. The enhancement of the critical speed is due to the improvement of the dynamic stiffness and the damping coefficients.
4. The designed spiral-grooved hybrid air spindle and an ordinary hybrid spindle was tested on the vibration in both axial and radial directions. The experiments showed results consistent with the calculated critical speed and verified the effect of groove structure on promoting the stability of a hybrid air spindle.

## Figures and Tables

**Table 1:** Bearing geometric parameters and operating parameters in this study.

Bearing Geometric Parameters and Operating Parameters	Value
Bearing diameter (D)	19.01 mm
Bearing length (L)	34.813 mm
Radius clearance (c)	21.5 μm
Orifice diameter (d)	0.12 mm
Column number of feeding orifices	2
Number of orifice on each column	10
Axial distance between orifice and bearing end face (L_1_)	12.7 mm
Groove depth (g_d_)	10 μm
Groove angle (β_g_)	π/6
Groove length (g_l_)	12.7 mm
Groove width ratio (α = w_g_/(w_r_ + w_g_))	0.5
Supplied pressure(P_s_)	6 bar

**Table 2:** Natural frequency of spiral-grooved hybrid air spindle at different speeds.

Rotation Speed (r/min)	Second-Order fs (Hz)	Third-Order fs (Hz)	Fourth-Order fs (Hz)	Fifth-Order fs (Hz)	Sixth-Order fs (Hz)	Seventh-Order fs (Hz)
10,000	617.67	1687	2134.1	3469.9	4285.1	4600
30,000	617.67	1704.3	2160.5	3484.3	4285.1	4610.7
50,000	617.67	1724	2185	3496.4	4285.1	4618.5
70,000	617.67	1739.3	2202.6	3504	4285.1	4623.3
90,000	617.67	1751.2	2216.3	3509.3	4285.1	4626.9
110,000	617.67	1759.9	2226.3	3513	4285.1	4629.5
130,000	617.67	1767.5	2235.3	3515.7	4285.1	4632
150,000	617.67	1773.5	2242.5	3518.4	4285.1	4634

**Table 3:** Dynamic stiffness, dynamic damping, critical perturbation frequency, critical mass and critical speed of a spiral-grooved hybrid air spindle under different eccentricities.

e	0.2	0.4	0.6	0.8
k_xx_ (N/μm)	1.980	0.459	−0.189	1.939
k_xy_ (N/μm)	2.028	0.568	−0.147	2.018
k_yx_ (N/μm)	2.099	0.738	−0.063	2.190
k_yy_ (N/μm)	2.185	0.999	0.093	2.468
d_xx_ (Ns/mm)	0.484	0.484	0.487	0.495
d_xy_ (Ns/mm)	0.080	0.080	0.077	0.066
d_yx_ (Ns/mm)	−0.145	−0.158	−0.185	−0.235
d_yy_ (Ns/mm)	0.497	0.513	0.558	0.659
w_c_ (r/min)	131,854.5	118,225.5	99,147	14,694
m_c_ (g)	873.2	3623.1	8037.5	13,473.3
W_1st_ (r/min)	58,496	54,026	51,890	49,137
W_2nd_ (r/min)	-	-	-	-

**Table 4:** The difference between the numerical model and experimental results under critical speed.

Shaft I	Critical Speed (r/min)	Shaft II	Critical Speed (r/min)
Numerical model (first order)	39,746	Numerical model (first order)	57,221
Experimental results (first order)	42,500	Experimental results (first order)	59,863
Difference	6.48%	Difference	4.41%
Numerical model (second order)	139,896	Numerical model (second order)	-
Experimental results (second order)	137,000	Experimental results (second order)	-
Difference	2.12%	Difference	-

## Data Availability

Not applicable.

## Nomenclature

C_d_	Discharge coefficient
d	Orifice diameter (mm)
e	Eccentricity ratio
f	Approximate solution
$\bar{f}$	Pressure square function
$\bar{f_{a}}$, ${\bar{f}}_{d}$	Dimensionless square atmosphere and downstream pressure
$\bar{h}$	Dimensionless air film thickness
H	Dimensionless groove depth (H = gd/c)
K_xx_, K_yy_, K_z_	Translational stiffness
K_θx_, K_θy_	Angular stiffness
L	Bearing length (mm)
$m_{r}$	Actual rth mass flow rate (kg/s)
$m_{t}$	Theoretical mass flow rate (kg/s)
$\bar{p}$	Dimensionless air film pressure
$p_{a}$	Atmosphere pressure
$p_{s}$	Supply pressure
P_0_	Dimensionless air film pressure in steady state
u v	Velocity components in the x and z directions (m/s)
$W_{x}$, $W_{y}$	Moment in x- and y- direction (N)
$\bar{x}$, $\bar{z}$	Dimensionless coordinates
λ	Perturbation frequency ratio
η	Air dynamic viscosity
$\xi_{i}$	Delta function
ρ	Air density (kg/m^3^)
$\rho_{a}$	Air density at atmosphere pressure (kg/m^3^)
Λ	Compressibility number
$\Lambda_{x}$, $\Lambda_{z}$	Dimensionless bearing numbers in the x- and z- direction
κ	Specific heat ratio of air
